# Supplementary material for: Deletion of wfs1 Impairs Oligodendrocyte Precursor Cells Dorsal Distribution and Myelination Through the wfs1-hmgcs1 Axis in Zebrafish
Source: Biology (Basel). 2026 Aug 16;15(16):1402. doi: 10.3390/biology15161402 (PMC13510151; doi:10.3390/biology15161402)
Supplement: Supplementary file 1 [file biology-15-01402-s001.zip › File S1-Transcriptome sequencing data and differentially expressed genes (DEGs) in wfs1-- zebrafish at 5 dpf/2.KEGG/wfs1KOvsWT/UP/src/dre00520.html]

dre00520


- dre:321224

- Up regulated genes

321224(1.93576834540418)

- dre:321224

- Up regulated genes

321224(1.93576834540418)

- dre:100885772

- Up regulated genes

100885772(1.27467501747957)

- dre:100885772

- Up regulated genes

100885772(1.27467501747957)

- dre:321224

- Up regulated genes

321224(1.93576834540418)

- dre:321224

- Up regulated genes

321224(1.93576834540418)

- dre:321224

- Up regulated genes

321224(1.93576834540418)

- dre:751668

- Up regulated genes

751668(2.48606367672347)

- dre:751668

- Up regulated genes

751668(2.48606367672347)

- dre:321224

- Up regulated genes

321224(1.93576834540418)

- dre:100885772

- Up regulated genes

100885772(1.27467501747957)

Close
